# Intersection of Pediatric Hypertension and Cardiovascular-Kidney-Metabolic Disease

**DOI:** 10.1007/s11906-026-01375-6

**Published:** 2026-06-23

**Authors:** Jessica O’Keeffe, Christina Kingshill, Jack Tompson, Andrew M. South, Carissa M. Baker-Smith

**Affiliations:** 1 Sidney Kimmel Medical College of Thomas Jefferson University, Philadelphia, PA USA; 2 Nemours Preventive Cardiology Program, Nemours Cardiac Center, Wilmington, Delaware USA; 3 Nemours Cardiac- Center for Research and Innovation, Nemours Children’s Health, Wilmington, Delaware USA; 4 Wake Forest University School of Medicine, Winston-Salem, NC USA; 5 Division of Public Health Sciences, Department of Epidemiology and Prevention, Wake Forest University School of Medicine, Winston-Salem, NC USA; 6 Department of Surgery-Section of Surgical Sciences, Wake Forest University School of Medicine, Winston-Salem, NC USA; 7 Nemours Cardiac Center, Nemours Children’s Health, 1600 Rockland Road, Wilmington, Delaware 19803 USA

**Keywords:** AAP, american academy of pediatrics, ABPM, ambulatory blood pressure monitoring, ACEi, angiotensin-converting enzyme inhibitor, AHA, american heart association, ARB, angiotensin II receptor blocker, ASCVD, atherosclerotic cardiovascular disease, BAT, brown adipose tissue, BNP, brain natriuretic peptide, CHD, congenital heart disease, CKD, chronic kidney disease, CKM, cardiovascular-kidney-metabolic syndrome, CPG, clinical practice guideline, CRS, cardiorenal syndrome, DASH, dietary approaches to stopping hypertension, DOHaD, developmental origins of health and disease, EGF, epidermal growth factor, ER, endoplasmic reticulum, FFA, free fatty acids, GFR, glomerular filtration rate, HTG, hypertriglyceridemia, IL-18, interleukin-18, KIM-1, kidney injury molecule-1, MAP, mean arterial pressure, NGAL, neutrophil gelatinase-associated lipocalin, NHANES, national health and nutrition examination survey, NT-proBNP, N-terminal pro-B-type natriuretic peptide, PAI-1, plasminogen activator inhibitor 1, PVAT, perivascular adipose tissue, RAAS, renin-angiotensin-aldosterone system, ROS, reactive oxygen species, SGLT2, sodium-glucose co-transporter 2 protein inhibitor, SNS, sympathetic nervous system, SRP, negative social risk profile, T2DM, type 2 diabetes mellitus, TG, triglycerides, TNFR-1, -2, tumor necrosis factors1 and 2, UACR, urine albumin-creatinine ratio, VSMC, vascular smooth muscle cell, WAT, white adipose tissue, WCH, white coat hypertension

## Abstract

**Purpose of Review:**

The purpose of this review is to describe the intersection between pediatric hypertension and advancing stages of cardiovascular, kidney metabolic syndrome in children and adolescents.

**Recent Findings:**

Cardiovascular-kidney-metabolic syndrome is highly prevalent in the pediatric population. The onset of CKM in childhood is influenced by the presence of antenatal risk factors such as maternal hypertensive disorders of pregnancy. Advancing stages of CKM in children and adolescents are strongly influenced by food insecurity and social determinants of health that impact the risk for childhood obesity. Activation of the renin-angiotensin-aldosterone system is a key mediator in the relationship between antenatal risk, early life course exposure and hypertension in children and adolescents. Effective strategies for slowing the rate of advancement of CKM staging in children and adolescents require attention to early course factors that influence the development of hypertension and obesity. Likewise, management strategies and therapeutic interventions that address these factors are critical to mitigating CKM stage advancement.

**Summary:**

Although hypertension is a component of the CKM framework, the presence of hypertension in children and adolescents drives higher CKM staging. Together, hypertension and higher CKM staging are associated with increased atherosclerotic cardiovascular disease risk. Social factors, including access to healthy foods and attention to early life course nutrition are critical strategies to improving CKM and hypertension related outcomes in children and adolescents.

## Introduction: Definition of Cardiovascular-Kidney-Metabolic Disease

**Fig. 1:**
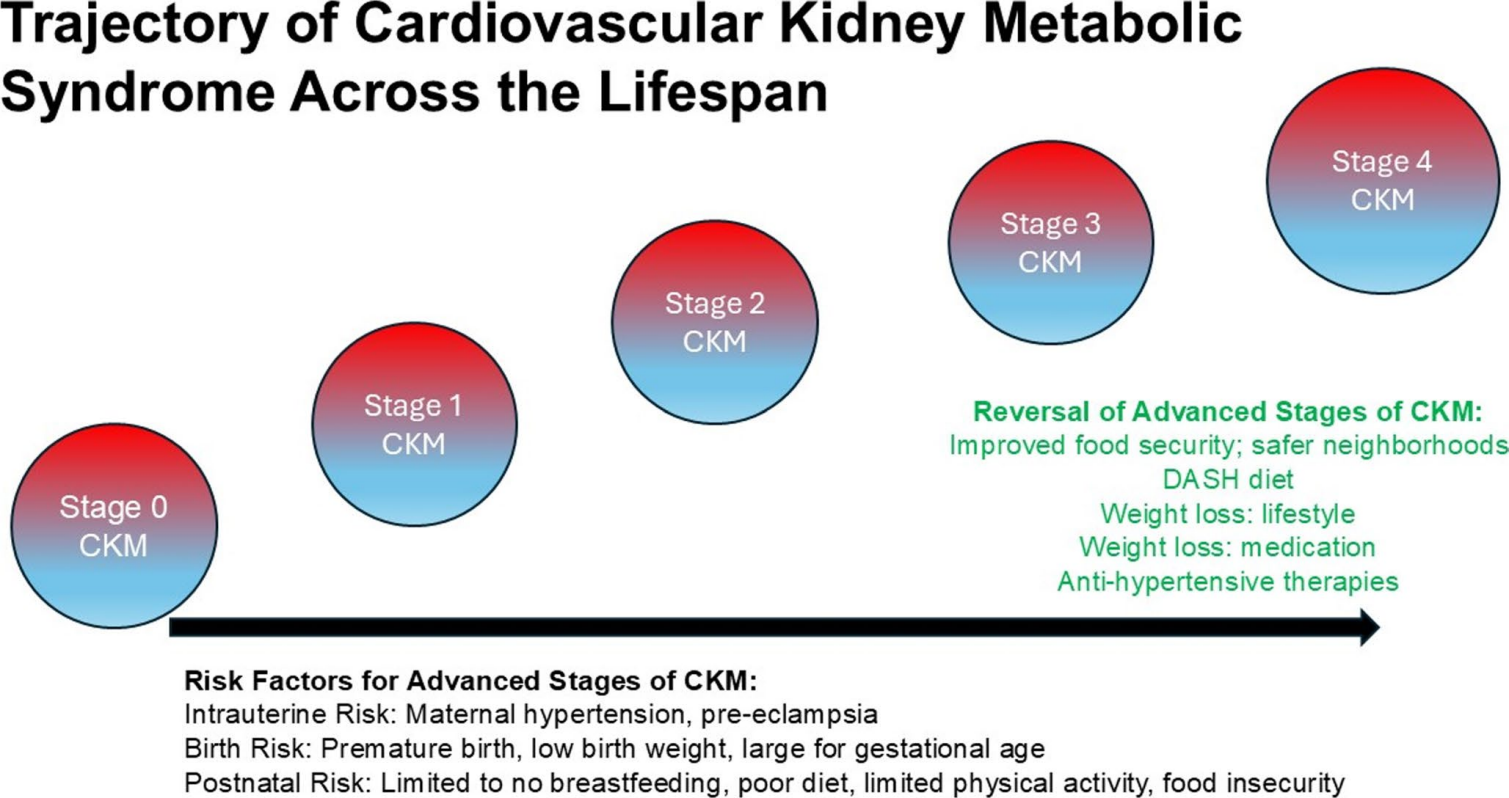
Cardiovascular Kidney Metabolic Syndrome Stages Legend: Cardiovascular kidney metabolic syndrome consists of 4 stages. Hypertension is a part of stage 2 but can also contribute to accelerated CKM staging. Efforts to improve blood pressure should be holistic and include weight loss. However, to fully reduce the risk for CKM, efforts are needed to reduce intrauterine risk for disease as well as to address comorbidities such as chronic kidney disease (CKD)

The American Heart Association (AHA) cardiovascular-kidney-metabolic (CKM) health framework, first described in 2023, represents an interrelated group of risk factors and conditions that lead to advanced atherosclerotic cardiovascular disease (ASCVD) (Fig. 1). These interrelated risk factors and conditions include obesity, insulin resistance, type 2 diabetes mellitus (T2DM), dyslipidemia, and chronic kidney disease (CKD). CKM confers a greater risk for ASCVD-related morbidity and mortality more than any individual risk factor or condition alone [1–4].

CKM is classified into four stages: stage 0, the absence of risk factors; stage 1, the presence of excess or dysfunctional adiposity; stage 2, the onset of metabolic risk factors for ASCVD such as hypertriglyceridemia (HTG), insulin resistance, T2DM, CKD, and hypertension; stage 3, evidence of subclinical atherosclerosis; and stage 4, clinical ASCVD, including the absence of chronic kidney failure (4a) or the presence of kidney chronic failure (4b).

## Section 1: Prevalence of CKM

CKM stages 1–4 are highly prevalent across the lifespan, and risk factors may emerge early in childhood. Although advanced stages (3–4) are uncommon in pediatric populations, data from the National Health and Nutrition Examination Survey (NHANES) 2017–2020 indicate that 37% of adolescents (12–18 years old) meet criteria for CKM syndrome stage1 and 7% meet criteria for CKM syndrome stage 2 [5]. Among adults, 89.4% have CKM stage 1–4, while only 10.6% have CKM stage 0. Nearly 64% of adults have poorer CKM stages (2–4), and it is very rare for a single condition to occur in isolation [3, 4, 6, 7].

Unfortunately, data regarding the prevalence of CKM in children aged 2–11 years remain limited. However, evidence suggests that risk factors for CKM may develop early in life, and even prior to conception [8, 9]. Maternal antenatal factors (e.g., factors that occur during the period spanning conception to birth) may influence fetal structural and metabolic development, potentially programming long-term cardiovascular risk through activation of the renin-angiotensin-aldosterone system (RAAS) [9]. The developmental origins of health and disease (DOHaD) prenatal programming framework posits that adverse early-life conditions (e.g., impaired lactation) may predispose individuals to hypertension and ASCVD [10, 11]. A recent cohort study found a positive association between maternal and offspring blood pressure and other CKM biomarkers, potentially due to RAAS dysfunction [12]. Similarly, premature birth is associated with high blood pressure and vascular stiffness also related to early physiologic changes involving RAAS [13, 14].

## Section 2: Excess or Dysfunctional Adiposity and Blood Pressure

CKM pathogenesis begins with the presence of disproportionate or dysfunctional adipose tissue [1, 2] Adipose tissue consists of white adipose tissue (WAT), which stores energy as triglycerides (TG) and free fatty acids (FFA), and brown adipose tissue (BAT), which facilitates thermogenesis. In normal-weight individuals, WAT constitutes 20–25% of body mass. In obesity, WAT expansion contributes to hypertension and metabolic dysregulation. BAT, while important for temperature regulation, does not significantly mitigate weight gain in obesity [15].

Adipose tissue functions as an endocrine organ, meaning it sends signals throughout the bloodstream and impacts distant target cells throughout the body with some paracrine function (e.g., signaling from cell to cell). The exocrine signals include the release of cytokines (e.g., tumor necrosis factor (TNF-alpha), interleukin-6 (IL-6), plasminogen activator inhibitor (PAI-1), fibrinogen, and hormones such as leptin, angiotensinogen, and adiponectin [15]. Adipose tissue surrounding blood vessels, referred to as perivascular adipose tissue (PVAT), can directly impact the blood vessel wall through paracrine signaling, and has been shown to modulate vascular function and peripheral resistance [15].

It is estimated that 60–75% of primary hypertension risk is attributable to excess body weight” [15]. In persons with obesity, adipocyte hypertrophy and hyperplasia are accompanied by infiltration of macrophages and lymphocytes, leading to the release of proinflammatory adipokines and vasoconstrictors. These processes contribute to endothelial dysfunction, vascular smooth muscle cell (VSMC) proliferation and migration, and vascular inflammation [16].

Under normal physiologic conditions (e.g., normal weight), adipose tissue maintains a balanced adipokine profile that supports vascular homeostasis through endocrine and paracrine pathways. However, in the presence of excess adipose tissue, adipokine expression becomes dysregulated. In addition, increased adiposity is also associated with expanded circulating blood volume, potentially mediated by activation of the sympathetic nervous system (SNS) and the renin–angiotensin system (RAS) [16].

Physical compression of the kidneys by fat deposition within and surrounding the kidneys, as well as excess visceral adiposity, may further contribute to the development of elevated blood pressure. Additional potential mechanisms contributing to obesity-related hypertension include hyperinsulinemia via enhanced sodium retention; increased angiotensin II production and subsequent vasoconstriction driven by adipose-derived angiotensinogen [17]; heightened adrenergic stimulation; oxidative stress; endothelial dysfunction; stimulation of VSMC growth due to increased FFAs; and leptin-mediated activation of the SNS [18].

## Section 3: Metabolic Disease and Blood Pressure

Historically, metabolic syndrome has been considered a “cluster” of conditions that includes abdominal obesity, HTG, low levels of high-density lipoprotein, and insulin resistance. However, there has been significant heterogeneity in how metabolic syndrome has been defined across studies and guidelines. More recently, it has been reframed not as a singular disease entity, but as a spectrum of overlapping systemic disorders encompassing obesity, diabetes, chronic kidney disease (CKD), and cardiovascular disease (CVD), collectively termed cardio-kidney-metabolic (CKM) syndrome.

In addiiton, there is significant variability in pediatric reference values, particularly for waist circumference and glucose metabolism thresholds,, further complicating diagnostic consistency [19]. Despite these definitional differences, common underlying pathophysiologic mechanisms—such as insulin resistance, chronic inflammation, neurohormonal activation, and endothelial dysfunction—are shared across this spectrum and have important implications for blood pressure regulation in the setting of metabolic dysfunction. 

The prior concept of metabolic syndrome is now incorporated into CKM syndrome. Multiple models have been created to explain the interplay between cardiovascular disease, CKD and the metabolic components of CKM syndrome. CKM pathophysiology involves hemodynamic and neurohormonal dysregulation, including SNS and RAAS activation, enhanced release of chemical mediators (e.g., nitric oxide, prostaglandins, endothelin, etc.), and oxidative stress [20]. Hyperglycemia leads to oxidative stress through excessive influx of glucose into cells, which increases mitochondrial superoxide production.” This process results in excess generation of reactive oxygen species (ROS) along with diminished antioxidant defense mechanisms” [21].

Loss of redox homeostasis creates a proinflammatory and profibrotic milieu that disrupts insulin metabolic signaling, impairs endothelial-mediated vasorelaxation, and contributes to structural and functional cardiovascular and renal abnormalities. These maladaptive processes are increasingly recognized as central to the progression of hypertension within the cardiorenal metabolic phenotype” [21]. Activation of the RAAS further elevates blood pressure through sodium and water retention and associated vasoconstriction. Additional CKM-related mechanisms include T2DM-associated endoplasmic reticulum (ER) stress, abnormal calcium handling, and chronic inflammation [20]. When cardio and renal systems have been under this stress for several years, those organs undergo changes that affect their ability to function [20].

At a more basic level, overnutrition, defined as excessive consumption of foods high in fat and carbohydrates, along with elevated aldosterone and angiotensin II levels, contributes to insulin resistance. Angiotensin II further drives oxidative stress and inflammation and increases circulating free fatty acids. These processes impair pancreatic beta-cell function and promote the development and progression of diabetes [22].

## Section 4: Advanced CKM Staging and Primary Hypertension in Children and Adolescents

Advanced stages of CKM are associated with hypertension. Hypertension plays not only a central role in CKM pathophysiology, but also in the advancement of CKM staging. Subclinical ASCVD, or CKM stage 3, is identified through either elevation in cardiac biomarkers (e.g., brain natriuretic peptide (BNP), N-terminal pro-B-type natriuretic peptide (NT proBNP), high sensitivity troponins) or evidence of abnormal cardiovascular structure and function in the absence of symptoms [1, 2]. High blood pressure may contribute to glomerular injury, arterial stiffening, and adverse cardiac remodeling [1, 2]. These effects are further amplified by metabolic abnormalities, such as obesity and insulin resistance, which exacerbate hypertension by increasing SNS activity, impairing natriuresis, and upregulating RAAS signaling [1, 2]. These changes drive myocardial fibrosis and left ventricular hypertrophy, ultimately compromising left ventricular function [23].

Further evidence of the impact of hypertension on advancing CKM stages can be found in a recent analysis from the Jackson Heart Study that estimated the heart rate (HR) of developing stage 4 CKM (e.g., ASCVD event) among participants with stages 2 or 3 CKM. The authors found that among persons with hypertension and CKM stages 1, 2 or 3, the HR for progressing to CKM stage 4 were 1.4, 7.5, and 26.6 per 1000 person-years respectively [24]. In another study, post-hoc analysis of secondary data from a cluster randomized trial indicated that improved blood pressure control (e.g., targeting a blood pressure less than 130/80 mmHg) was associated with reduced cardiovascular events across all CKM stages [25].

## Section 5: CKM Stages, Chronic Kidney Disease and Renovascular Hypertension

Hypertension and CKM occur concomitantly in children and adolescents [26]. The classification of any hypertension as CKM syndrome stage 2 underscores the significant cardiovascular burden associated with elevated blood pressure across the lifespan [1, 23]. Compared with primary hypertension, renovascular hypertension and CKD confer an even greater risk for future ASCVD (Fig. 1) [23].

Unlike in adults, the etiology of chronic kidney disease (CKD) in children and adolescents is not primarily driven by diabetes. Instead, pediatric CKD commonly arises from conditions such as congenital heart disease (CHD), congenital anomalies of the kidneys and urinary tract (CAKUT), and chronic neurohormonal activation of the renin–angiotensin–aldosterone system (RAAS) [26, 27].

Among children with CHD and heart failure, elevated venous pressures—rather than reduced renal perfusion—are now recognized as a key mechanism linking cardiovascular disease to acute and, ultimately, chronic kidney disease (i.e., cardiorenal syndrome) [26]. Additionally, pediatric CKD may develop in the setting of persistent RAAS activation. Increased levels of angiotensin II contribute to myocardial remodeling, fibrosis, and hypertrophy, changes that are characteristic of CKM stage 3. Furthermore, higher urine albumin-to-creatinine ratio (UACR), an established marker of renal and cardiovascular risk, is significantly associated with increased odds of CKM stage 3 or 4 disease [27, 28].

The American Heart Association (AHA) CKM advisory does not provide specific recommendations for kidney function testing in children and adolescents with stage 2 or higher CKM. In clinical practice, serum creatinine remains the primary marker used to estimate kidney function; however, reduced muscle mass in pediatric populations may result in overestimation of glomerular filtration rate (GFR) [27]. Cystatin C, an alternative biomarker that is independent of muscle mass, is increasingly used but may be influenced by non-renal factors such as steroid use, inflammation, age, and adiposity [26, 29]. Measurements of UACR and estimated GFR (eGFR) are routinely used in clinical care [27].

Emerging biomarkers may offer additional utility in the assessment of CKD within the CKM framework. These include neutrophil gelatinase-associated lipocalin (NGAL), interleukin-18 (IL-18), kidney injury molecule-1 (KIM-1), epidermal growth factor (EGF), α₁-microglobulin, and inflammatory markers such as tumor necrosis factor receptors 1 and 2 (TNFR1 and TNFR2) [27]. Although research on these biomarkers is ongoing, they hold potential as diagnostic and prognostic tools for evaluating CKM progression in youth, particularly those with hypertension, when used alongside traditional measures such as eGFR and UACR [27].

Regardless of the measure used to assess renal function, trends in renal function should be monitored longitudinally to assess CKM progression across the lifespan [1, 2]. Risk factors for CKM may originate in utero, including alterations in kidney development and function [11].

Maternal hypertensive disorders of pregnancy are common pregnancy-related complications [30] Pregnancy associated hypertension, even when it occurs among women who deliver at term, is associated with a higher systolic blood pressure in the offspring [31]. Early-life programming can impact all of the cardiovascular-related tissues, most notably the kidneys, heart, vasculature, and adiposity, with RAAS activity playing a central role in these processes [9, 32, 33]. Infants born large for gestational age exhibit a 25% higher risk of adolescent hypertension [13].

Intrauterine exposures also influence the development of CKD. Like hypertension, evidence suggests that kidney disease may be programmed during fetal development [22]. Premature birth is associated with incomplete nephrogenesis, a higher incidence of acute kidney injury, and an increased risk for future kidney disease, including proteinuria and reduced GFR [13]. These processes may be mediated by the RAAS, as experimental models demonstrate that RAAS deficiency results in reduced nephron number and underdeveloped renal structures [22]. Conversely, hypertension may be exacerbated by local RAAS activation that occurs in the setting of CKD.^22^ In pediatric populations, angiotensin II and aldosterone induce hypertension and vascular remodeling [26].

## Section 6: CKM Staging and White Coat Hypertension

White coat hypertension (WCH) is defined as an elevated blood pressure reading in the clinical setting, but normal blood pressure outside [34]. WCH is a diagnosis that may precede hypertension and may also intersect with CKM staging. A small retrospective longitudinal study of pediatric patients referred for ambulatory blood pressure monitoring (ABPM) found that the prevalence of WCH was 80% and that 61% of pediatric patients 12-17years with WCH progressed to having an abnormal ABPM phenotype within 14 months [35].

ABPM is considered the gold standard for diagnosing WCH. Children and adolescents with WCH who undergo repeat blood pressure evaluation within six months are at risk of developing an abnormal ABPM phenotype, specifically ambulatory prehypertension or ambulatory hypertension [1, 35]. The American Academy of Pediatrics Clinical Practice Guideline for Screening and Management of High Blood Pressure in Children and Adolescents (AAP CPG) recommends repeating ABPM every 2–3 years in children with WCH [34]. In the context of CKM disease, for patients with white coat hypertension, follow up blood pressure monitoring is recommended to screen for any progression to abnormal blood pressure readings, especially if the patient has other metabolic risk factors such as hypertriglyceridemia or diabetes.

Current CKM stage 2 management emphasizes modification of risk factors to prevent further progression, specifically through lifestyle interventions and adherence to established blood pressure guidelines [1]. When a patient is diagnosed with WCH, the Dietary Approaches to Stopping Hypertension (DASH) diet is commonly recommended to help prevent progression to true hypertension. In the adult population, greater adherence to the DASH diet was associated with a lower risk of higher CKM stages [36]. When patients are diagnosed with WCH, the recommendation of a DASH diet may help prevent transition to overt hypertension or more advanced CKM stages with greater cardiovascular and renal involvement.

Nutrition represents a critical component of CKM prevention and management, yet food insecurity remains common among pediatric populations. Studies have shown 14–28% of adolescents lack consistent access to food [5]. Patients with WCH should therefore undergo social determinants of health screening to better understand their capacity to adhere to the DASH diet recommendations and other recommended lifestyle interventions. Understanding social and environmental barriers to adherence enables clinicians to provide more tailored care Table 1.

**Table 1:** Prevention and Management of Hypertension According to CKM Stage in Children and Adolescents

CKM Stage	Description	Methods for preventing and treating hypertension	Goals
0	No risk factors	Prevention: DASH diet, endorse regular physical activity. Maintain an optimal weight/BMI Avoid smoking/vaping Obtain and maintain adequate sleep Address social factors when possible	Maintain optimal health parameters [37].
1	Excess or dysfunctional Adiposity	Prevention (of hypertension, CKD, metabolic syndrome): DASH diet, exercise Treatment: (options) Lifestyle: diet, exercise + Pharmacologic interventions: GLP1RA, topiramate, topiramate/phentermine use if *≥* 12 years Surgical interventions: T/c Bariatric surgery if *≥* 12 years	BMI < 85th percentile
2	Hypertriglyceridemia, hypertension, diabetes, metabolic syndrome or moderate-to-high risk CVD	Treatment: (options) Lifestyle change: diet, exercise Pharmacologic interventions: GLP1RA, topiramate, topiramate/phentermine use if *≥* 12 years Sodium-Glucose Transport Protein 2 (SGLT2) inhibitors Hypertriglyceridemia: t/c/ fenofibrate, omega-3 fatty acid, HMG CoA reductase inhibitor (e.g., statin) Antihypertensive therapy: angiotensin converting enzyme inhibitor (ACEi), angiotensin receptor blocker (ARB), +/ diuretic	BP < 95th percentile or < 130/80 if no CKD BP < 90th percentile or < 120/<80 if CKD or DM
3	Subclinical cardiovascular disease (CVD)	Treatment: (options) Lifestyle change: diet, exercise Pharmacologic interventions: GLP1RA, topiramate, topiramate/phentermine use if *≥* 12 years Sodium-Glucose Transport Protein 2 (SGLT2) inhibitors Hypertriglyceridemia: t/c/ fenofibrate, omega-3 fatty acid, HMG CoA reductase inhibitor (e.g., statin) Antihypertensive therapy: angiotensin converting enzyme inhibitor (ACEi), angiotensin receptor blocker (ARB), +/ diuretic	BMI < 85th percentile BP < 95th percentile or < 130/80 if no CKD BP < 90th percentile or < 120/<80 if CKD or DM Non-HDL-C < 145 mg/dL

Legend: CKM stage description, recommended prevention strategies and goals of intervention

## Section 7:Social Factors: Food Insecurity and CKM Staging

Independent of age, demographic characteristics and lifestyle factors, a negative social risk profile (SRP) is associated with more advanced stages of CKM. Among adults, 20 to 79 years of age, a lower, more negative SRP is associated with a 34% greater odds of CKM stage 1 (OR 1.34, 95% CI: 1.06–1.70), a 100% greater odds of CKM stage 2 (OR 2.03, 95% CI: 1.59–2.58), a 428% greater odds of CKM stage 3 (OR 5.28, 95% CI: 3.29–8.47), and a nearly 500% greater odds of CKM stage 4 (OR 5.97, 95% CI: 4.20–8.49) [38]. Higher stages of CKM are more prevalent among adults with less education (e.g., less than high school vs. college or higher education), unemployment (vs. employment), uninsured health status (vs. insured status), and those who rent their home (vs. own a home) [39]. The presence of a single adverse childhood experience is associated with 5% greater HR for ASCVD (HR 1.05; 95% CI: 1.04-0.1.12, *p* < 0.001), while exposure to *≥* 4 adverse childhood experiences is associated with a 35% higher risk (HR 1.35; 95% CI: 1.10–1.67) [40].

Advanced stages of CKM disproportionately impact persons who reside within high-risk areas. Limited access to healthy foods is a risk for higher stages of CKM. Children and adolescents with food insecurity are at greatest risk for advanced stages of CKM. Analysis of roughly 1,700 youth participants in the NHANES study demonstrated an association between food insecurity and more advanced stages of CKM [5]. This data highlights the importance of considering and addressing social factors when identifying risks and solutions for more advanced stages of CKM.

## Section 8: Management and Treatment: At the Intersection of CKM and Hypertension

ASCVD primordial/primary prevention is most effective when the interconnected spectrum of risk factors associated with CKM is treated holistically. Thus, the CKM framework encourages clinicians and researchers to view these interconnected conditions as a unified syndrome-such that improvements in one may lead to improvements in another in an additive or even multiplicative manner.

Lifestyle interventions are recommended at all stages of CKM, including stage 0 (i.e., primordial prevention). The “progressive staging” of CKM highlights the need not only for a multidisciplinary approach to management, but also prevention of more advanced stages (i.e., primary prevention) [41]. In the presence of dysfunctional adiposity, even before the onset of hypertension, adoption of the DASH diet and lower salt intake has been shown to reduce blood pressure in persons with and without hypertension [42]. In the absence of this choice of diet, higher stages of CKM may develop [36].

Hypertension is a key risk factor within the CKM syndrome framework [1, 2]. For youth, upon developing CKM conditions such as hypertension, regardless of obesity, the AHA calls for annual screening for hyperlipidemia, hypertension, and hyperglycemia, as well as measuring kidney function via UACR and eGFR [1, 2]. In this way, CKM guidelines focus on preventing hypertension through obesity management and then implementing more detailed testing when hypertension arises. 

When hypertension develops, implementation of the DASH diet is recommended. If blood pressure does not adequately improved with lifestyle modification alone, then antihypertensive therapy is recommended [34]. In both early- and late-stage CKM syndrome, the RAAS is a key mediator of cardiac, metabolic, and renal outcomes, and drugs such as RAAS inhibitors should be considered for the management of stage 2 or greater CKM syndrome in pediatric populations [1, 43]. Angiotensin-converting enzyme inhibitors (ACEi) and angiotensin receptor blockers (ARBs) are the preferred therapy in the presence of CKD, independent of race [32]. However, dosing may need to adjusted in order to maintain the nephroprotective effects of this therapy [43].

For youth with CKD or renovascular hypertension, more conservative blood pressure control is recommended (goal 24-hour MAP < 50th percentile by ABPM) [34, 44]. Overall, CKD is a risk enhancer for ASCVD in persons with CKM and early management, including during childhood and adolescence, is essential for improved long-term outcomes. More advanced stages of CKM and CKD require timely subspeciality referral and management [41].

The sodium-glucose cotransporter 2 protein (SGLT2) inhibitors have been shown to reduce cardiovascular-related mortality and heart failure hospitalizationsin patients regardless of the presence or absence of diabetes [45]. Among individuals with CKD, these agents have been found to be useful in adults. Current adult guidelines recommend use of SGLT2 inhibitors in persons with CKD to prevent further deterioration in kidney function [46, 47]. However, additional clinical trials are currently in progress to assess whether SGLT2 inhibitors have a positive impact on cardiovascular and kidney outcomes in the children and adolescents [48–50].

Finerenone, a nonsteroidal mineralocorticoid receptor antagonist, has undergone safety, efficacy and tolerability testing in adults (e.g., DEDELIO-DKD and FIGARO-DKD) and is currently being investigated in pediatric patients (e.g., FIONA) [51]. According to data from the FIDELIO-DKD study, among 5734 adult diabetic patients, use of finerenone in persons with CKD 3–4 lead to a lower risk for CKD progression (e.g., kidney failure) and cardiovascular events (e.g., death, nonfatal myocardial infarction, stroke and hospitalization) [52]. It has yet to be determined whether or not pediatric studies will yield a similar result.

Beyond management of hypertension and CKD, stage 0 CKM requires longitudinal monitoring, including tracking weight, blood pressure, HbA1c, and lipid levels. According to a Cochrane meta-analysis of 172 randomized controlled trials, interventions including physical activity reduce BMI modestly in children and adolescents with overweight or obesity, whereas diet alone has limited effect [37, 53]. Similarly, a meta-analysis of 72 randomized clinical trials found that exercise interventions improve cardiorespiratory fitness in children and adolescents with overweight or obesity [54]. High-intensity interval training may be particularly beneficial [55]. Reductions in BMI in childhood may portend a similar ASCVD risk to children with normal BMI, if normalization of the BMI can occur before adulthood [56].

Stage 1 CKM is defined as having excess or dysfunction adipose tissue, obesity, or increased BMI > 25 kg/m2 [1, 2]. The treatment goal in stage 1 is to decrease BMI and address the adipose tissue. Lifestyle interventions remain first-line therapy and include reducing intake of ultra-processed foods, increasing adherence to a DASH-style dietary pattern, and promoting regular physical activity. However, clinicians must consider that not all persons will have equal access to healthy foods or places to safely exercise. Thus, interventions should consider not only individual-level factors, but interpersonal, community and social policy level factors when promoting the treatment and primordial/primary prevention of advanced stages of CKM [1, 5, 57].

Lifestyle intervention alone may be insufficient. The development of obesity is multifactorial, influenced by physiologic mechanisms, early-life exposures (e.g., maternal obesity and early introduction of solid foods), genetic predisposition, and socioeconomic and environmental factors [58]. FDA-approved therapies for weight loss in children and adolescents may be considered. These agents include: topiramate/phentermine [59], orlistat [60], and the glucagon-like-peptide-1 receptor agonists (GLP-1RA)s, liraglutide [61] and semaglutide [62, 63].

Among children and adolescents with stage 2 CKM, the treatment goal is to prevent progression to subclinical cardiovascular disease (stage 3) and clinical ASCVD (stage 4). In addition, in adults, GLP1-RAs demonstrated renal and cardioprotective benefits [64, 65]. In pediatric populations with type 2 diabetes or obesity, GLP-1 RAs improve glycemic control, weight, and cardiometabolic outcomes [66].

Management of hypertriglyceridemia in children and adolescents includes lifestyle modifications (e.g., moderate to vigorous physical activity), management of overweight and obesity when indicated, and evaluation for secondary causes. In moderate hypertriglyceridemia (fasting TG of 401-885 mg/dL) that do not respond to lifestyle intervention, treatment with fenofibrate or omega-3 fatty acids may be considered. An HMG-CoA reductase inhibitor (e.g., statin) may be considered if the non-HDL-C is *≥* 145 mg/dL [67].

## Section 9: Policies to Improve CKM Prevention, Detection and Treatment in the Pediatric Population policy and Systems/Community Solutions to Address, Prevent and Treat CKM

There is currently limited evidence evaluating the impact of policy interventions on improving CKM (cardiovascular-kidney-metabolic) health. However, a growing body of literature highlights the critical role of social drivers of health in shaping CKM outcomes [5, 68, 69]. For example, children with food security demonstrate approximately 40% lower odds of developing early-stage CKM (stages 1–2) [5, 68]. Among adults, factors such as income instability, disadvantaged neighborhood environments, lower educational attainment, and adverse physical and psychological conditions are associated with increased odds of advanced CKM syndrome [69].

These findings suggest that policies aimed at improving food security, enhancing neighborhood conditions, expanding job access, promoting economic stability, and addressing social and psychological environments may help prevent progression to more advanced CKM stages. Furthermore, increased efforts to improve access to healthy foods may benefit not only adults but also children and adolescents, supporting healthier trajectories across the lifespan.

## Section 10: Future Directions

Given the early onset of risk factors for CKM, including in some cases, pre-conception and antenatal, efforts to address the intergenerational risk for CKM are needed. Newer and validated methods for assessing kidney function to enhance our ability to better detect more advanced stages of CKM in children and adolescents are needed. Newer trials and therapies are also needed to most effectively manage the early stages of CKM. Addressing implementation challenges, such as ensuring equitable access to care and access to effective therapies for all at risk, are needed. Finally, additional data regarding the potential benefit of kidney-protective therapies such as ACEi/ARB, SGLT2i and finerenone deserves to be further investigated.

## Conclusions

The combination of CKM factors into one framework allows for earlier detection of CVD and CKD and shifts the focus from isolated disease management to a more comprehensive, systems-based approach to health. This holistic approach to care is likely to have greater ASCVD primordial/primary prevention benefit. Although hypertension is a component of CKM, it also drives progression to more advanced CKM stages and must be addressed early—beginning in the preconception period and continuing through the antenatal period, childhood, and adolescence.

## Key References

- Ndumele CE, Rangaswami J, Chow SL, et al. Cardiovascular-Kidney-Metabolic Health: A Presidential Advisory From the American Heart Association. Circulation. 2023;148(20):1606-1635.
  - ○ Pivotal manuscript and the first to describe the cardiovascular-kidney-metabolic health framework.
- Chen Y, Wu X, Long T, Jiang Y, Wang M, Lv Z, Hou H, Li Z, Liu M. Prevalence and Mortality Association of Different Stages of Cardiovascular-Kidney-Metabolic Syndrome. JACC Adv. 2025 Jun;4(6 Pt 2):101843.
  - ○ Prospective cohort study that describes the prevalence of CKM stages 1-4 among 110,933 participants of the UK Biobank
  - ○ Nearly 90% of UK Biobank participants meet criteria for CKM syndrome (stages 1-4); 80% with poor CKM stages (2-4).
  - ○ Describes the relationship between CKM stage and cardiovascular (CVD) disease specific mortality rates.Higher stages of CKM syndrome are strongly associated with increased risks of both all-cause mortality and CVD-specific mortality.
- Lei L, Li J, Ding W, Wang W, Yu Y, Pu B, Peng Y, Zhang L, Guo Y. Associations of Cardiovascular-Kidney-Metabolic Syndrome with Premature Mortality and Life Expectancies in US Adults: A Cohort Study. Cardiorenal Med. 2025;15(1):484-495. doi: 10.1159/000546618. Epub 2025 Jul 1. PMID: 40592307; PMCID: PMC12215152.
  - ○ Cross sectional analysis of data from the  National Health and Nutrition Examination Survey (NHANES) (1999-2018), including 18,350 US adults aged 20-79 years.
  - ○ Describes the prevalence of CKM stages 0-4 in adults and the associated risk for all-cause mortality: The prevalence of CKM stages 1, 2, 3, and 4 is 23.1%, 53.6%, 3.6%, and 6.7%, respectively.
  - ○ Compared with CKM stage 0, individuals in stage 4 have a markedly higher risk of all-cause mortality and lost years of life at age 50 years.
- Baker-Smith CM, Gauen AM, Petito LC, Khan SS, Allen NB. Prevalence of Cardiovascular-Kidney-Metabolic Stages in US Adolescents and Relationship to Social Determinants of Health. J Am Heart Assoc. 2025;14(10):e040269
  - ○ Cross-sectional analysis of NHANES data form 1774 survey 12–17-year-olds to describe the prevalence of CKM stages in adolescents and to describe the factors driving higher CKM staging in children and adolescents.
  - ○ The prevalence of CKM stages in children and adolescents include: 37% and 7% for CKM stages 1 and 2, respectively. Food security is associated with a 40% lower odds of CKM stage 1 to 2 after adjustment for all sociodemographic factors.
- Alexander BT, South AM, August P, Bertagnolli M, Ferranti EP, Grobe JL, Jones EJ, Loria AS, Safdar B, Sequeira-Lopez MLS; American Heart Association Council on the Kidney in Cardiovascular Disease; Council on Cardiovascular and Stroke Nursing; Council on Cardiovascular Radiology and Intervention; Council on Hypertension; and Council on Lifestyle and Cardiometabolic Health. Appraising the Preclinical Evidence of the Role of the Renin-Angiotensin-Aldosterone System in Antenatal Programming of Maternal and Offspring Cardiovascular Health Across the Life Course: Moving the Field Forward: A Scientific Statement From the American Heart Association. Hypertension. 2023 May;80(5):e75-e89. 
  - ○ Scientific statement from the American Heart Association (AHA) that summarizes the role of the renin-angiotensin-aldosterone system in mediating the relationship between antenatal hypertensive disorders of pregnancy and later offspring hypertension.
  - ○ This statement also highlights that disparities in access to adequate prenatal care is a significant risk factor for the disproportionate prevalence of hypertensive disorders of pregnancy among subpopulations.
- Robinson CH, Hussain J, Jeyakumar N, et al. Long-Term Cardiovascular Outcomes in Children and Adolescents With Hypertension. JAMA Pediatr. 2024;178(7):688-698.
  - ○ Population-based, retrospective, matched cohort study conducted from 1996 to 2022 assessing the long-term associated risk of major adverse cardiac events (MACE) (e.g., cardiovascular death, stroke, hospitalization for acute MI or unstable angina, or coronary intervention) among children with hypertension.
  - ○ Children diagnosed with hypertension have double the associated risk of developing MACE compared with controls without hypertension.
- Palaniappan LP, Allen NB, Almarzooq ZI, Anderson CAM, Arora P, Avery CL, Baker-Smith CM, Bansal N, Currie ME, Earlie RS, Fan W, Fetterman JL, Barone Gibbs B, Heard DG, Hiremath S, Hong H, Hyacinth HI, Ibeh C, Jiang T, Johansen MC, Kazi DS, Ko D, Kwan TW, Leppert MH, Li Y, Magnani JW, Martin KA, Martin SS, Michos ED, Mussolino ME, Ogungbe O, Parikh NI, Perez MV, Perman SM, Sarraju A, Shah NS, Springer MV, St-Onge MP, Thacker EL, Tierney S, Urbut SM, Van Spall HGC, Voeks JH, Whelton SP, Wong SS, Zhao J, Khan SS; American Heart Association Council on Epidemiology and Prevention Statistics Committee and Stroke Statistics Committee. 2026 Heart Disease and Stroke Statistics: A Report of US and Global Data From the American Heart Association. Circulation. 2026 Mar 3;153(9):e275-e906.
  - ○ 2026 of the global prevalence of cardiovascular disease.
- Shahid I, Philip J, Avenatti E, et al. Lifestyle Interventions in Cardiovascular-Kidney-Metabolic Syndrome JACC: Advances Expert Panel. JACC Adv. 2025;4(6 Pt 2):101788.
  - ○ Review article that describes evidence from randomized clinical trials for specific lifestyle interventions across CKM syndrome stages.

## Data Availability

No datasets were generated or analysed during the current study.
